# Perception and Cognition of Cues Used in Synchronous Brain–Computer Interfaces Modify Electroencephalographic Patterns of Control Tasks

**DOI:** 10.3389/fnhum.2015.00636

**Published:** 2015-11-23

**Authors:** Luz María Alonso-Valerdi, Francisco Sepulveda, Ricardo A. Ramírez-Mendoza

**Affiliations:** ^1^Brain-Computer Interfaces (BCI) Group, School of Computing Science and Electronic Engineering, University of Essex, Colchester, UK; ^2^Escuela de Ingeniería y Ciencias, Tecnológico de Monterrey – Campus Ciudad de México, Mexico City, Mexico

**Keywords:** brain–computer interface, motor imagery, classification accuracy, electroencephalographic patterns, human factors

## Abstract

A motor imagery (MI)-based brain–computer interface (BCI) is a system that enables humans to interact with their environment by translating their brain signals into control commands for a target device. In particular, synchronous BCI systems make use of cues to trigger the motor activity of interest. So far, it has been shown that electroencephalographic (EEG) patterns before and after cue onset can reveal the user cognitive state and enhance the discrimination of MI-related control tasks. However, there has been no detailed investigation of the nature of those EEG patterns. We, therefore, propose to study the cue effects on MI-related control tasks by selecting EEG patterns that best discriminate such control tasks, and analyzing where those patterns are coming from. The study was carried out using two methods: standard and all-embracing. The standard method was based on sources (recording sites, frequency bands, and time windows), where the modulation of EEG signals due to motor activity is typically detected. The all-embracing method included a wider variety of sources, where not only motor activity is reflected. The findings of this study showed that the classification accuracy (CA) of MI-related control tasks did not depend on the type of cue in use. However, EEG patterns that best differentiated those control tasks emerged from sources well defined by the perception and cognition of the cue in use. An implication of this study is the possibility of obtaining different control commands that could be detected with the same accuracy. Since different cues trigger control tasks that yield similar CAs, and those control tasks produce EEG patterns differentiated by the cue nature, this leads to accelerate the brain–computer communication by having a wider variety of detectable control commands. This is an important issue for Neuroergonomics research because neural activity could not only be used to monitor the human mental state as is typically done, but this activity might be also employed to control the system of interest.

## Introduction

A brain–computer interface (BCI) is a system that enables humans to interact with their environment by translating their brain signals into control commands for a device of interest (Graimann et al., 2010). The mechanism of a BCI system fundamentally consists of two steps: (1) detecting and decoding the user intentions for controlling the system and (2) maintaining a continuous user-system communication. The *user intentions* of controlling a BCI system are changes in the user brain signals that are regulated through control tasks. A control task can be based on exogenous or endogenous paradigms (Jackson and Mappus, 2010). Particularly, the endogenous paradigm is based on the quantification of brain oscillations that are modulated via cognitive tasks such as motor imagery (MI). The *user-system communication* is established by a control interface. A control interface can be synchronous or asynchronous (Hassanien and Azar, 2015). In a synchronous interface, the user-system communication is allowed only in fixed time windows; whereas in an asynchronous interface, the user initiates the communication with the system at will. Both synchronous (Obermaier et al., 2003; Leeb et al., 2006; Maeder et al., 2012; Bamdadian et al., 2014) and asynchronous (Scherer et al., 2007; Galán et al., 2008; Lotte et al., 2008; Tseng et al., 2015) systems have been developed over the past few years. For real-world applications, the prototyping of asynchronous systems is preferred because these allow users to interact naturally with their environment. The relevance of synchronous systems cannot, however, be ignored, even in real applications. The cueing process facilitates the early and accurate detection of the user control tasks, despite the user ability for modulating his/her brain signals. This, in turn, raises confidence, persistency, and autonomy in the users toward the mastery of BCI skills. Furthermore, the identification of MI onset allows to analyze prior and post periods, which have been associated with the improvement of BCI performance and the recognition of the user cognitive state (Maeder et al., 2012; Bamdadian et al., 2014; Gutierrez et al., 2015).

As brain signals are modulated by neural networks that modify their degree of synchronization according to the sensory–cognitive input, it is not surprising that control tasks (particularly those based on MI) contain much more information than only that related to the user intention of controlling the system (Kropotov, 2010). MI-related control tasks are a source of information that has been exploited not only to generate control commands for a target device, but also to enhance BCI performance, to predict classification accuracy (CA), or to determine the user mental state. For example, Pfurtscheller and Neuper (2001), and then Obermaier et al. (2003), reported that left- and right-hand MIs were correctly discriminated as early as 250 ms after the onset of a specific visual cue. They attributed the early discrimination to the cue properties, concluding that the control tasks were the result of conscious (MI) and unconscious (visual stimulation) processes over the sensory–motor area of the brain. Furthermore, in a later and more detailed study, Pfurtscheller et al. (2008) found that distinct short-lasting brain patterns appeared within a time window of about 500–750 ms after cue onset. Those brain patterns produced different features for different imaginary movements (hands and feet), facilitating and accelerating the discrimination of MI-related control tasks in naïve subjects. Another example is the study carried out by Grosse-Wentrup and Scholkopf (2012) in which high gamma range (55–85 Hz) between two fronto-parietal networks were used to predict BCI performance on a trial-to-trial basis. Additional and important examples are two studies, respectively, undertaken by Maeder et al. (2012) and Bamdadian et al. (2014). Those researchers demonstrated that the user performance in classical synchronous BCIs can be predicted by quantifying the modulation of the brain signals on pre-cue stages because they reflected somehow the user cognitive state. Finally, and more recently, Scheel et al. (2015) found that visual and auditory cues provoked significant differences of the peak amplitude of movement-related cortical potentials in synchronous BCIs. They also found that potentials from the auditory-cue paradigm had a wider spatial distribution than those from the visual cue.

Overall, all aforementioned studies support the view that brain patterns extracted from MI-related control tasks can provide much more information than that used to control a target device. In particular, the cue effects on MI-related control tasks have been studied. Researchers in the field have shown that both perception (e.g., sensory–cognitive processing of the cue) and cognition (e.g., imaginary motor activity) are reflected on the brain signals wherefrom BCI control tasks are extracted. Studying the influence of human factors on BCI control tasks may help to design a more versatile human–machine interaction for this type of systems because of the active (e.g., extraction of control commands for manipulating a target device) and passive (e.g., monitoring of the level of attention of an individual) use of the brain signals. This work could have further applications in Neuroergonomics, where neural activity is registered in order to monitor human mental state. Making use of neural activity in an active and passive way may be much more fruitful.

So far, it has been shown that brain patterns before and after cue onset can reveal the user cognitive state and enhance the discrimination of MI-related control tasks. However, there has been no detailed investigation of the nature of those brain patterns. We, therefore, propose to study the cue effects on MI-related control tasks by selecting the brain patterns that best discriminate such control tasks, and analyzing where those patterns are coming from in order to answer two questions:

(1) If different cues provoke significant changes on MI-related control tasks, can different cues improve BCI performance as Scheel et al. (2015) suggested in their research? and
(2) If MI-related control tasks are defined by motor activity *per se* and the cue in use, do brain patterns proceed from sources (recording sites, frequency bands, and time intervals) not only associated with motor activity, but also related to the sensory–cognitive processing of the cue?

The present study was conducted as follows. First, brain activity was registered by means of electroencephalography (EEG). Second, the frequently used stimulation modalities (SMs) for cueing in training sessions were applied. These were auditory (Nijboer et al., 2008) and visual (Boostani et al., 2007) stimuli. In addition, a bimodal cue (combination of auditory and visual stimuli) was included in the study because previous investigations in sensory encoding (Basar et al., 1999; Isoğlu-Alkaç et al., 2007) have shown that simultaneous presentation of auditory, visual, and somatosensory stimuli significantly enhances sensory responses. Third, as preparation and imagination of movements evoke similar neural desynchronization events over the sensory–motor areas (Neuper et al., 2006) and both of them are widely used as control task, the two motor activities were included in the study. Finally, given that brain oscillations occur in a wide range of EEG recording sites, frequency bands, and time intervals (Kropotov, 2010); brain patterns were analyzed using two methods: standard and all-embracing. The standard method was restricted to the well-established motor activity sources (Pfurtscheller et al., 2007), while the all-embracing method involved all the available EEG information.

## Materials and Methods

### Experimental Procedure

#### Participant Recruitment and General Instructions

Nine participants (four females and five males) took part in this study, which was previously authorized by the Ethics Committee of the University of Essex. All of them were aged between 28 and 41 years. None of them reported auditory impairments, seven of them had normal vision, and two of them had corrected-to-normal vision. Eight of the nine reported to be right-handed and only one was left-handed.

The participants were informed about the experimental procedure and signed a consent form. Only two of the nine had previously engaged in cognitive tasks related to imagination of movements. At the beginning of the experiment, every participant was carefully instructed as follows:

- Get ready to imagine the movement of the hand that indicates a track playing “left” or “right” (audio), an arrow pointing to left or right (visual), or both of them (bimodal).
- Imagine yourself opening and closing your formerly pointed hand as soon as you listen to an increasing tone, see a green bulb, or perceive both of them.
- Stop the MI process and relax as soon as you listen to a decreasing tone, see a red bulb, or perceive both of them.

#### Organization of the Experiment

**Figure 1:**
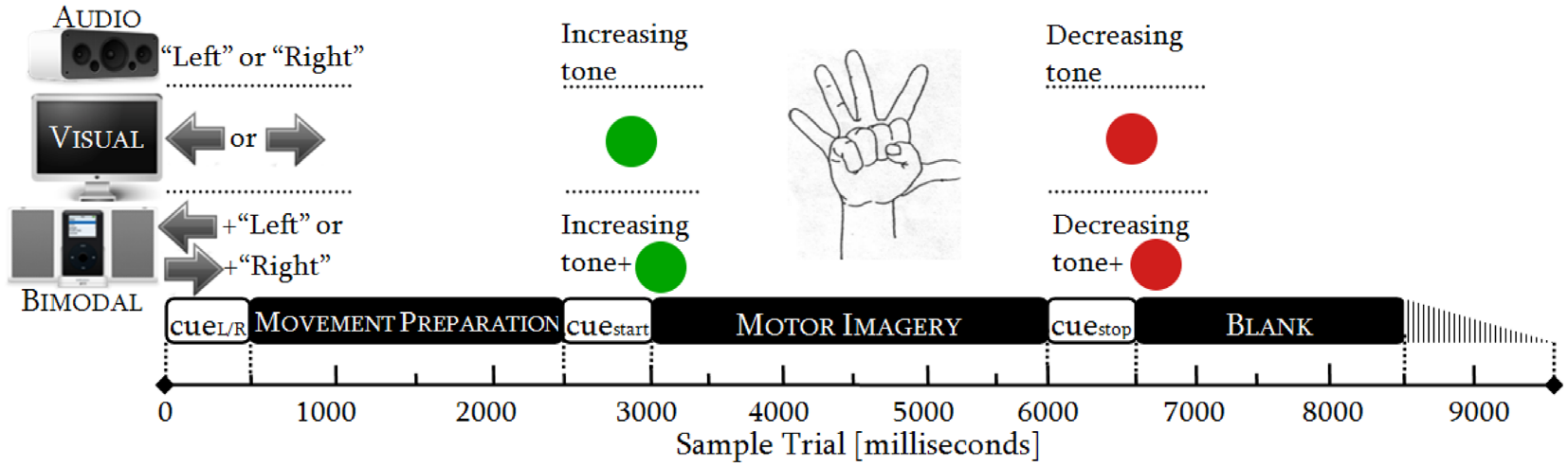
**Timing protocol: audio, visual, and bimodal stimuli for cueing MP and MI**.

In order to collect sufficient EEG data, the participants attended two sessions. The sessions lasted 48 min each and followed an identical procedure. Every session consisted of six runs and one run had 50 trials. One trial took from 8500 to 9500 ms (Figure 1), resulting in runs of ~8 min. Within each trial, there were three phases: MP (0–2500 ms), MI (2500–6000 ms), and relaxing (6000–8500 ± 1000 ms). In the latter phase, a random variation of 1000 ms was included to reduce expectation effects.

As there were three SMs (audio, visual, and bimodal) and both hands (left and right) were involved, there were six categories of trials: audio-left, audio-right, visual-left, visual-right, bimodal-left, and bimodal-right. Each of these categories was randomly presented 50 times and distributed over the six runs. We thereby obtained 12 conditions (six categories of trials × two control tasks) and one condition had 100 trials (2 sessions × 50 trials).

#### Timing Protocol

The duration of the cues was standardized to 500 ms in accordance with sensory recognition and reaction time studies (Teichner, 1954; Shelton and Kumar, 2010). The movement preparation (MP) was adjusted to 2000 ms, which is the necessary period to achieve readiness in the neural networks over the sensory–motor area (Jeannerod, 2006; Neuper et al., 2006). The MI was limited to 3000 ms, as is commonly done in synchronous BCIs. The relaxation span varied from 2000 to 3000 ms, guaranteeing a proper recovery of the longest desynchronization process, i.e., the alpha one (Pfurtscheller et al., 1996). See Figure 1.

#### EEG Data Collection

The EEG signals were recorded by means of Biosemi equipment (Amsterdam, The Netherlands), the integration of ActiveTwo system and ActiView software (Honsbeek et al., 1998). The ActiveTwo system was configured to acquire the signals within a bandwidth between DC and 400 Hz, and at a sampling frequency of 2048 Hz. The ActiView software was programed to decimate the signals at 512 Hz. Such configuration limited the effective digital bandwidth to 104 Hz by default.

**Figure 2:**
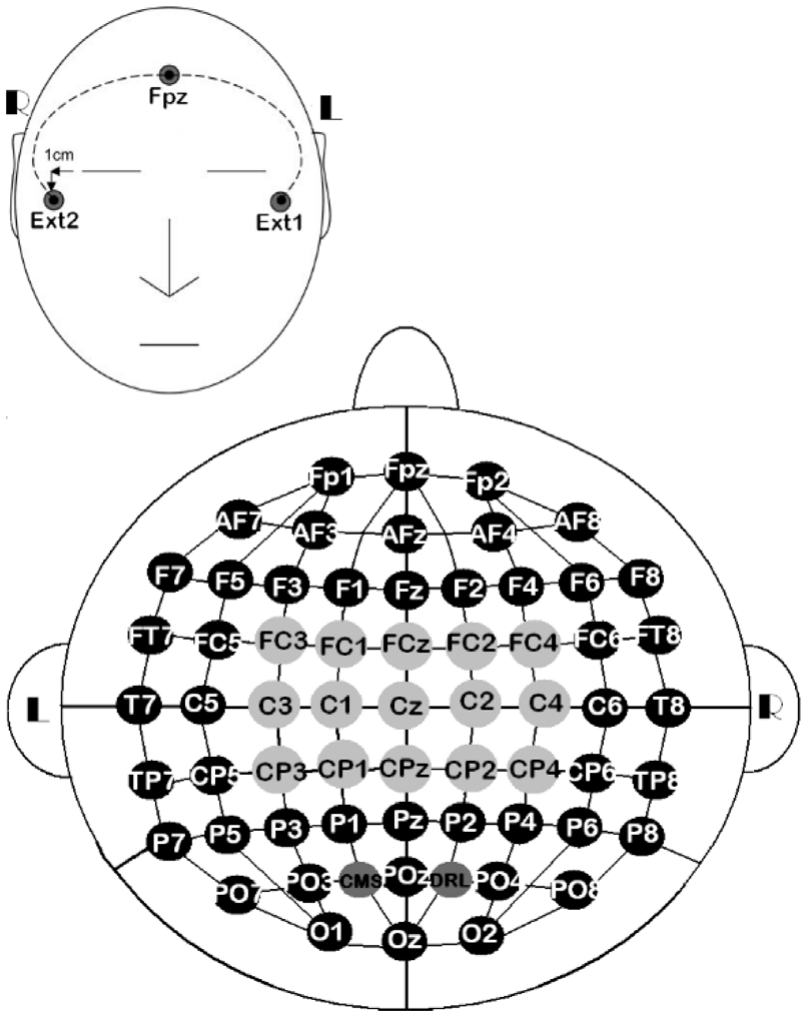
**10/10 EEG layout of 61 channels, along with three external electrodes**. All the EEG channels (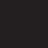, 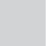) were used for the all-embracing method, 15 of them (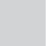) were used for the standard method, EOG_L_/EOG_R_ were used for recording eye movements, and M_R_ was used for referencing EEG and EOG signals.

The EEG signals were sensed via 61 active electrodes, plus driven-right-leg and common-mode-sense electrodes. The 61 active electrodes were mounted on a head-cap labeled as stated in the 10/10 system. The other two electrodes were only used for referencing electrically the ActiveTwo system, but they were not recorded. In addition, three external electrodes were included for recording the eye movements (EOG). Two of them (EOG_L_ and EOG_R_) were placed 1 cm below and above the lateral canthus of the left and right eyes, respectively. The third one was placed on the right mastoid (M_R_) for referencing EEG and EOG signals (Figure 2). At the end of the experiments, we gathered 18 datasets (9 participants × 2 sessions).

### Data Analysis

The datasets of one participant were excluded from the study. Those showed electrode-pop artifacts over the occipital area of the scalp. There were then 16 datasets for the study purposes.

#### Processing of Continuous EEG Data

**Figure 3:**
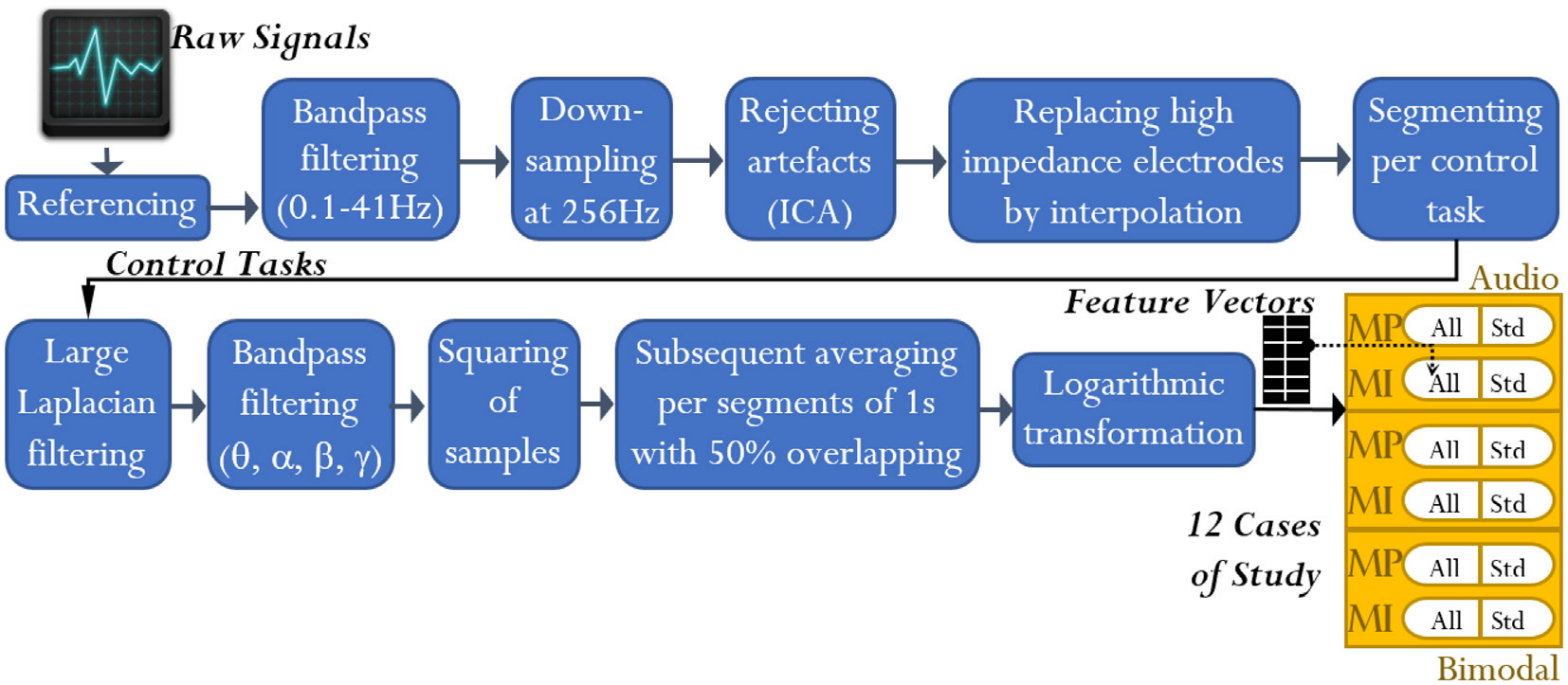
**EEG signal analysis: from raw signals to feature extraction**. At the end of the analysis, feature vectors for 12 cases of study resulted. Note that the term “All” refers to all-embracing method, and the term “Std” refers to standard method.

To attenuate the interference in the EEG channels, these were processed by using the open-access toolbox for electrophysiological signal processing, EEGLAB (Delorme and Makeig, 2004). First, every channel from each of the 16 datasets was processed as follows: (1) referencing against M_R_, (2) high-pass filtering at 0.1 Hz using a Butterworth filter of order 4, (3) low-pass filtering at 41 Hz using a Butterworth filter of order 7, and (4) down-sampling from 512 to 256 Hz. Second, every dataset was scanned to eliminate discontinuities and detect high-impedance electrodes. Up to three electrodes under this condition were detected per dataset. Third, independent component analysis was applied to each dataset for rejecting artifacts such as EOG and electrocardiography. Only EEG channels without high-impedance difficulties were involved in such analysis. EOG_L_ and EOG_R_ channels were used to identify all the independent components related to EOG activity. Finally, the EEG channels with high-impedance difficulties were replaced by interpolating their nearest neighboring channels as reported by Gargiulo et al. (2010). See Figure 3.

#### Processing of the Control Tasks (MP and MI)

The processing of the control tasks was carried out through the *miBCI* software^1^, package published by Alonso-Valerdi and Sepulveda (2015). From every EEG channel of the 16 datasets, the control tasks were extracted in line with the cue onset (Figure 1). The MP and MI were thus 2500 and 3500 ms long, respectively. Having obtained the EEG signals of interest, they were spatially filtered via large Laplacian in order to obtain more localized electrical activity (Dornhege et al., 2007).

#### Feature Extraction

It is well-established that MP and MI provoke neural desynchronization with peak power around 10 and 20 Hz (Neuper et al., 2009). As band power (BP) estimation has been validated as a stable and consistent method for quantifying EEG power changes due to motor activity (Neuper et al., 2005), this was selected as feature extractor. BP estimation was applied in line with the methods described below.

##### Standard Method

Previous investigations have empirically established the following criteria to effectively discriminate hand imaginary movements. First, 18 central recording sites have been validated as the maximum number of EEG channels for satisfactory classification (Ramoser et al., 2000). Second, narrow frequency bands around the maxima 10 and 20 Hz have been widely used in synchronous BCI systems (Pfurtscheller et al., 2007; Neuper et al., 2009). Third, it has become common practice to discard 1 s post-cue, wherein evoked potentials are typically detected (Boostani et al., 2007). With these criteria in mind, we laid down the *standard method*. This method was based on 15 central recording sites (Figure 2), four frequency bands, and EEG segments taking place 1 s post-cue. The frequency bands were established as follows: lower alpha (α_L_) from 8 to 10 Hz, upper alpha (α_U_) from 10 to 12 Hz, lower beta (β_L_) from 16 to 20 Hz, and upper beta (β_U_) from 20 to 24 Hz.

##### All-Embracing Method

EEG signals are regulated by brain oscillators that adjust their state of synchrony according to sensory (e.g., cue decoding) and cognitive (e.g., MP and MI) events. These oscillators are neural networks that enter into synchrony in a wide range of resonant frequencies (from 0 up to about 80 Hz) and over specific periods of time (Krause, 2003). In view of this fact, we extended the scope of the standard method by establishing the *all-embracing method*. This method was based on 61 recording sites (Figure 2), seven frequency bands, and whole trace of MP and MI. In addition to the previously mentioned bands, the following ones were also considered: lower theta (θ_L_) from 4 to 6 Hz, upper theta (θ_U_) from 6 to 8 Hz, and gamma (γ) from 39 to 41 Hz. These bands were included in the analysis on the basis of the following evidence. Theta band rhythms resonate at the frequency band 4–8 Hz and emanate from the frontal midline due to audio–visual information encoding, attention demands, memory retrieval, and cognitive load. Moreover, these rhythms enhance after practice on the cognitive tasks at hand. They are more prevalent when the subject is focused and relaxed, and prolonged activity is related to selective attention (Basar et al., 1999; Krause, 2003; Kropotov, 2010). The upper theta band (6–8 Hz) generally reflects levels of alertness (Pineda, 2005). On the other side, gamma band rhythms oscillate near 40 Hz during sensory encoding, perceptual–cognitive functions, and motor behaviors. These rhythms are phase-locked to the stimulus and short-lasting, and appear 100 ms post-stimulus in sensory–motor tasks (Pfurtscheller and Lopes da Silva, 1999; Ward, 2003; Altermaller et al., 2005).

Bearing in mind the criteria of standard and all-embracing methods, we can now briefly describe the feature extraction based on BP. The MP/MI signals were first filtered through Butterworth band-pass filters of order 7, with cut-off frequencies defined by the afore-stated bands. Afterwards, the signals were squared per sample and segmented by using time windows of 500 ms length with 50% overlapping rate. Finally, the resulting time segments (herein denoted by δ*_*n*_*) were averaged and logarithmically transformed (refer to Figure 3), obtaining nine features per MP signal and 13 features per MI signal.

By the standard method, there were 15 channels and 4 frequency bands under consideration. In addition, three time segments [δ_1_ (0–500 ms), δ_2_ (250–750 ms), and δ_3_ (500–1000 ms)] were discarded. Hence, vectors of 300 features for MP and vectors of 540 features for MI were obtained. By the all-embracing method, vectors of 3843 features for MP and vectors of 5551 features for MI were similarly obtained.

#### Feature Selection and Classification

After the feature extraction, there were 24 types of feature vectors that proceeded from three SMs, two control tasks, two hands, and two methods. These feature vectors were grouped by merging left and right MIs. Having obtained 12 different cases of study (Figure 3), Davies–Bouldin indexes (DBIs) were determined in each case to increasingly sort the corresponding features (Sepulveda et al., 2004; Kovács et al., 2005). DBI is a method for measuring the linear separability among *m* classes (Equation 1). This metric is based on comparing the similarity (*R*) among classes. Such similarity is determined by the class dispersion (*s*) and the distance (*d*) between centroids (Eq. 2). The class dispersion is the average distance between every element (τ) in the class and the centroid of the class (*v*). See Eq. 3. Thereby, the features within each vector were ranked from the most to the least suitable feature in terms of linear separability between two classes: left and right (Kovács et al., 2005). Note that smaller DBIs correspond to major linear separability.

(1) $$\text{DBI}=\frac{1}{m}\sum_{\text{i}=1}^{\text{m}}R_{\text{i}}$$

where $R_{\text{i}}=\underset{\begin{array}{l} j=1,...,m\\ i\neq j \end{array}}{\max}\left(R_{\text{ij}}\right)$ and *i* = 1,…,*m*

(2) $$R_{\text{ij}}=\frac{s_{\text{i}}+s_{\text{j}}}{d_{\text{ij}}}=\frac{s_{\text{i}}+s_{\text{j}}}{d\left(v_{\text{i}},v_{\text{j}}\right)}$$

(3) $$s_{\text{i}}=\frac{1}{{\text{T}}_{\text{i}}}\sum_{{}_{}}d\left(\tau ,v_{\text{i}}\right)$$

where *T*_i_ is the number of features in class *i*.

**Figure 4:**
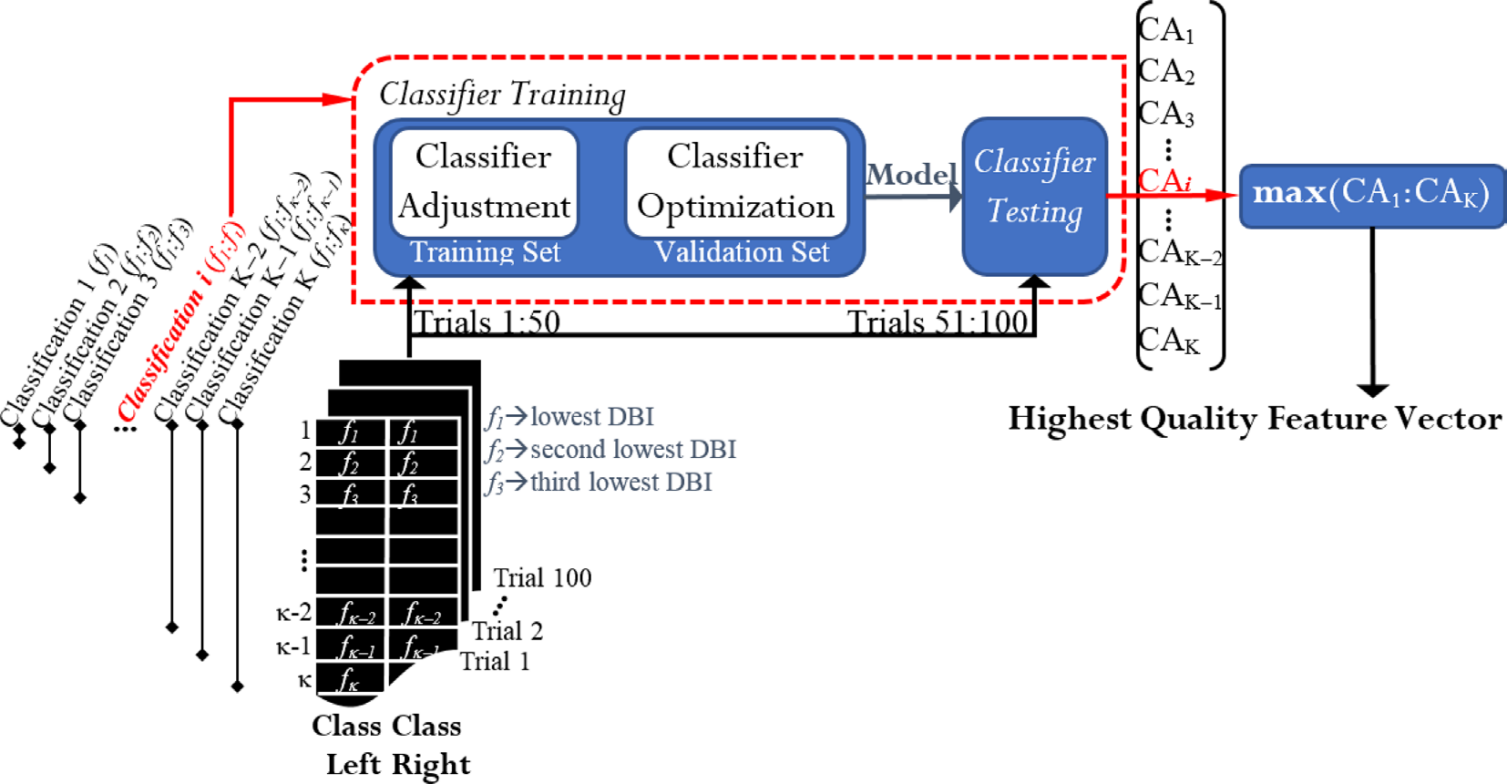
**Feature selection and classification: obtaining the highest quality feature vectors (HQFVs)**. For each case of study, a HQFV was obtained. A HQFV was the feature vector that reached the maximum CA and wherein each feature (*f*) was ranked according to DBI.

After ranking the features, a classification process took place in order to select the appropriate number of features that best discriminated between left and right. If there were two classes and κ denoted the total number of features in each vector, K classifications were run for each case of study (Figure 4). From the K resulting CAs, the feature vector yielding the maximum performance was selected from each case of study. Thereby, we obtained 12 feature vectors for every participant. They were called the highest quality feature vectors (HQFVs). Note that the term “maximum performance” refers to 1.5 times the interquartile range plus the upper quartile of the general distribution of all the CAs obtained at the end of the process. As a result, any peak value that was beyond the 99% of the distribution was discarded.

Every classification process^2^ was based on Fisher discriminant analysis (FDA) and consisted of two phases: training and testing. The 100 available trials per cluster were distributed half and half; that is, 50 trials (session 1) for training and 50 trials (session 2) for testing. The classifier was trained via 10-fold cross validation. That is, 50 training trials were split into a training set and a validation set. Through the validation set, the model was optimized by adjusting the regularization term that generally avoids overfitting problems due to the large number of features in use (Bishop, 2006). Once the classifier had been trained, this was tested by the rest of the trials and the percentage of the total number of correct predictions was estimated. The resulting CA in the testing phase was the parameter for acquiring the HQFVs. See Figure 4.

In this study, all statistical analyses were performed using the non-parametric method Kruskal–Wallis one-way ANOVA, and significance levels were set at 5%.

### Statistical Evaluation of the HQFVs

The features of the HQFVs proceeded from specific recording sites (e.g., C3, Cz, or C4), frequency bands (e.g., α_L_, α_U_, β_L_, or β_U_) and time windows (δ_n_). The origin of a feature in any of these three domains (location, frequency, and time) was referred as to *feature source*. On this basis, the HQFVs were statistically evaluated in accordance with those three domains and under two parameters: index of dispersion (ID) and *mode*.

The ID was calculated by using Eq. 4 and was an approach to quantify how spread a HQFV was over the feature sources in each domain. In Eq. 4, *k* is the number of feature sources in the domain of interest, *f_*i*_* is the number of occurrences of each feature source, and *N* is the total number of features in the HQFV under analysis (Norman and Streiner, 2008). Note that ID is 0 when all the features fall into one feature source. By contrast, it is 1 when the features are equally divided among the *k* feature sources.

(4) $$\text{ID}=\frac{k\left(N^{2}-\sum f_{i}^{2}\right)}{N^{2}\left(k-1\right)}$$

The *mode* was the central tendency of a HQFV, i.e., the most frequently occurring feature source in the domain at hand. Having gathered the *modes* of all the HQFVs, these were graphically represented via a 2D-histogram (*modal* distribution) for each domain. In every 2D-histogram, the number of occurrences of each *mode* (*f_*mode*_*) was normalized by diving *f_*mode*_* by *N*.

## Results

### Classification Accuracy of the HQFVs

**Figure 5:**
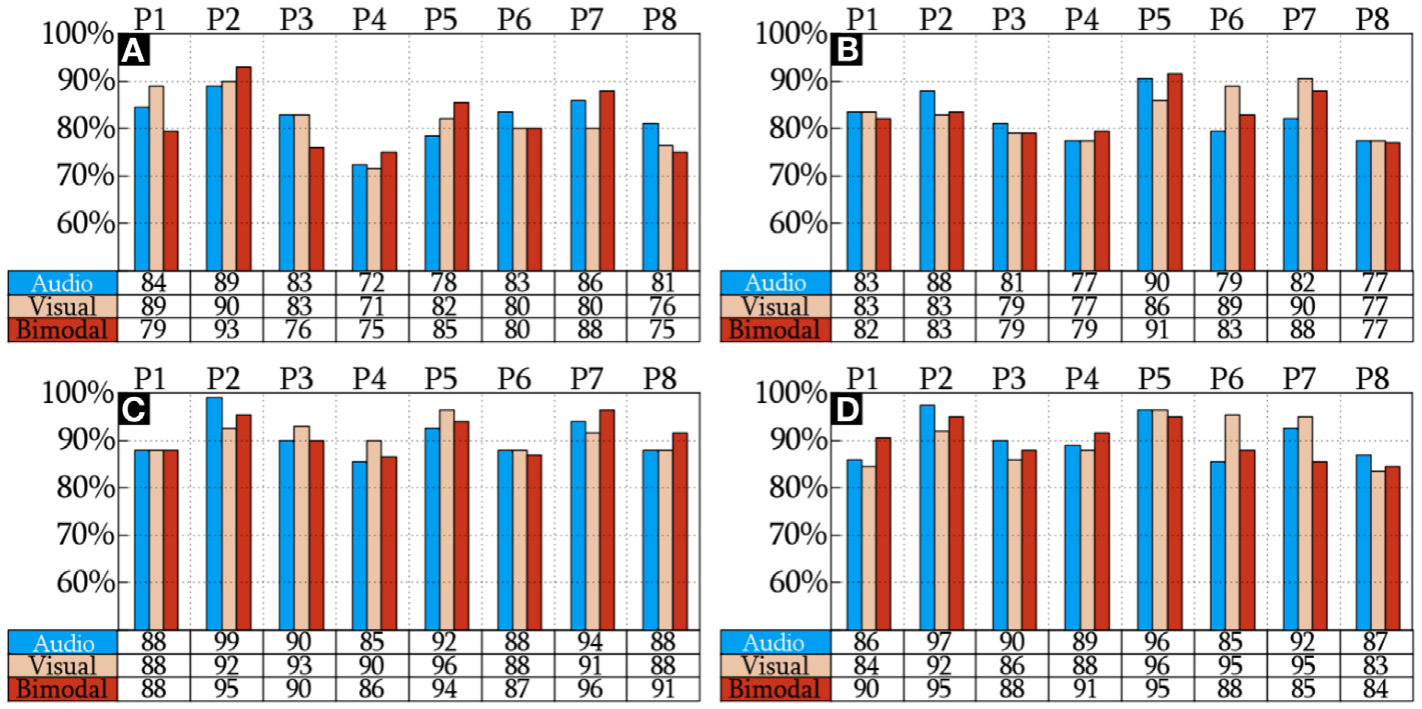
**CA of the HQFVs**. MP and MI analyzed using the standard method are illustrated in **(A,B)**, respectively. MP and MI analyzed using the standard method are shown in **(C,D)**, respectively.

The CAs reached by the HQFVs are arranged in Figure 5. This figure indicates that there is no significant difference of CAs among SMs (*p* = 0.935). The figure also indicates that there is a significant increase of CAs (*p* = 1.11 × 10^−16^) between standard and all-embracing methods for both control tasks (MP and MI) and the three SMs (audio, visual, and bimodal). Finally, the figure shows that CAs (*p* = 0.707) between MP and MI are comparable for the three SMs and the two methods in use.

### Index of Dispersion of the HQFVs in Location, Frequency, and Time

**Figure 6:**
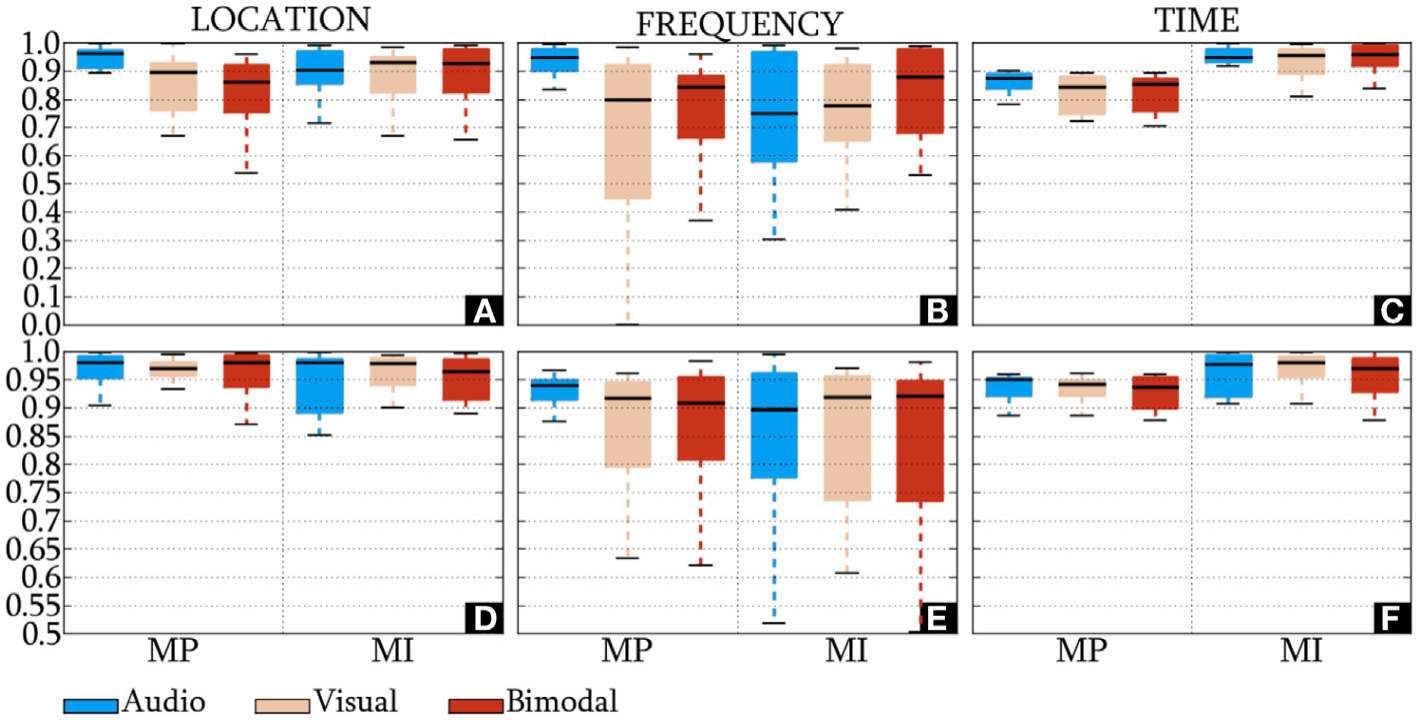
**IDs of the HQFVs**. For the standard method, the indexes of the HQFVs over feature sources in location, frequency, and time are depicted in **(A–C)**, respectively. For the all-embracing method, the indexes of the HQFVs over feature sources in location, frequency, and time are presented in **(D–F)**, respectively.

The IDs of the HQFVs, which were obtained from the standard method, are presented in Figures 6A–C. In location and time, the HQFVs are generally spread over all the feature sources showing IDs above 0.52 and 0.7, respectively. By contrast, IDs range between 0 and 1 in frequency. The IDs of the HQFVs, which resulted from the all-embracing method, are provided in Figures 6D–F. These IDs are above 0.85, 0.5, and 0.87 in location, frequency, and time, respectively. The statistical comparison of the IDs between both methods in location, frequency, and time resulted in the following *p*-values: 1.461 × 10^−9^, 0.049, and 4.767 × 10^−7^. Note that all the remarks mentioned in this section apply to MP and MI.

### Modal Distribution of the HQFVs in Location, Frequency, and Time

#### Standard Method

**Figure 7:**
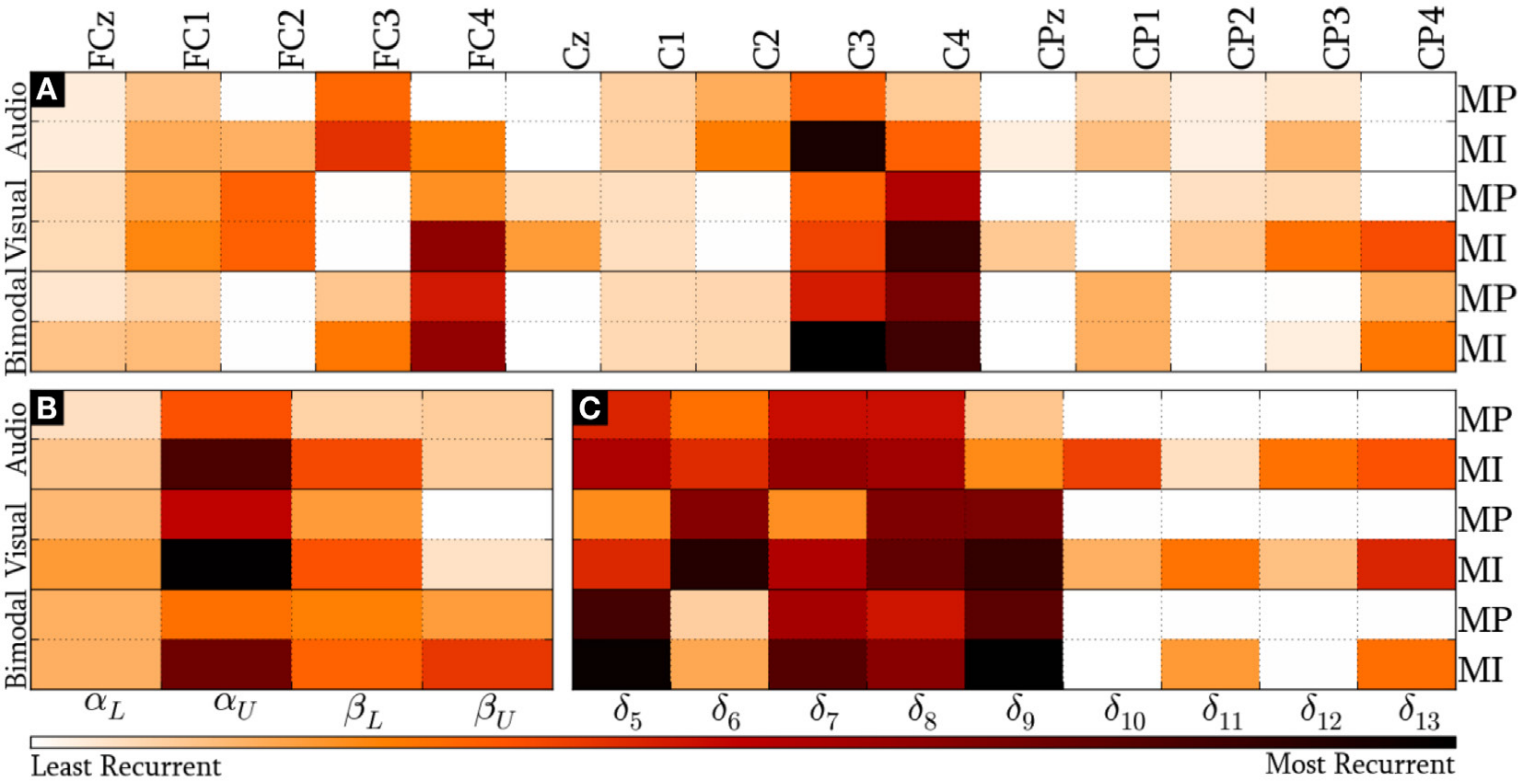
***Modal* distribution of the HQFVs that resulted from the standard method**. The *modal* distribution is illustrated in three domains: **(A)** 15 recoding sites, **(B)** 4 frequency bands, and **(C)** 5/9 time windows for MP/MI.

Figure 7 presents the *modal* distribution of the HQFVs over the following feature sources: (a) 15 recording sites, (b) 4 frequency bands, and (c) 5/9 time windows for MP/MI. With regard to the location domain, Figure 7A shows that *modes* from audio cues mainly tend toward FC3 and C3, while those from visual cues mostly tend to FC2, FC4 (only applicable for MI), and C4. *Modes* from bimodal cues are essentially distributed among FC3, C3, FC4, and C4. In all the cases, MI displays greater tendencies than MP.

We can see from Figure 7B that the overriding band for the three SMs is α_U_. In this case, the highest and the lowest tendencies are reached by *modes* from visual and bimodal cues, respectively. The MI control task shows a second dominant band. Such dominant band for *modes* from audio and visual cues is β_L_, while that for *modes* from bimodal cues is β_U_. The MP control task only shows a second dominant band for *modes* from bimodal cues, which is β_L_. As in the location domain, MI reveals stronger tendencies in comparison with MP.

Lastly, Figure 7C provides the *modal* distribution in time. Keeping in mind that MP only involved five time windows (from δ_5_ to δ_9_), we can see that *modes* from the three SMs are evenly distributed along most of them. Although MI involved the nine time windows, the *modes* from the three SMs are mostly distributed across δ_5_ and δ_9_ as well. In both cases, the major *modal* tendencies for audio, visual, and bimodal cues are, respectively, the following: δ_7_/δ_8_ (1500–2000 ms), δ_6_/δ_8_/δ_9_ (1250–2500 ms), and δ_5_/δ_9_ (1000–1500 ms and 2000–2500 ms). There is additionally a relevant *modal* distribution over δ_13_ for the three SMs in MI, regardless of the decreasing trend of the foregoing time windows.

#### All-Embracing Method

**Figure 8:**
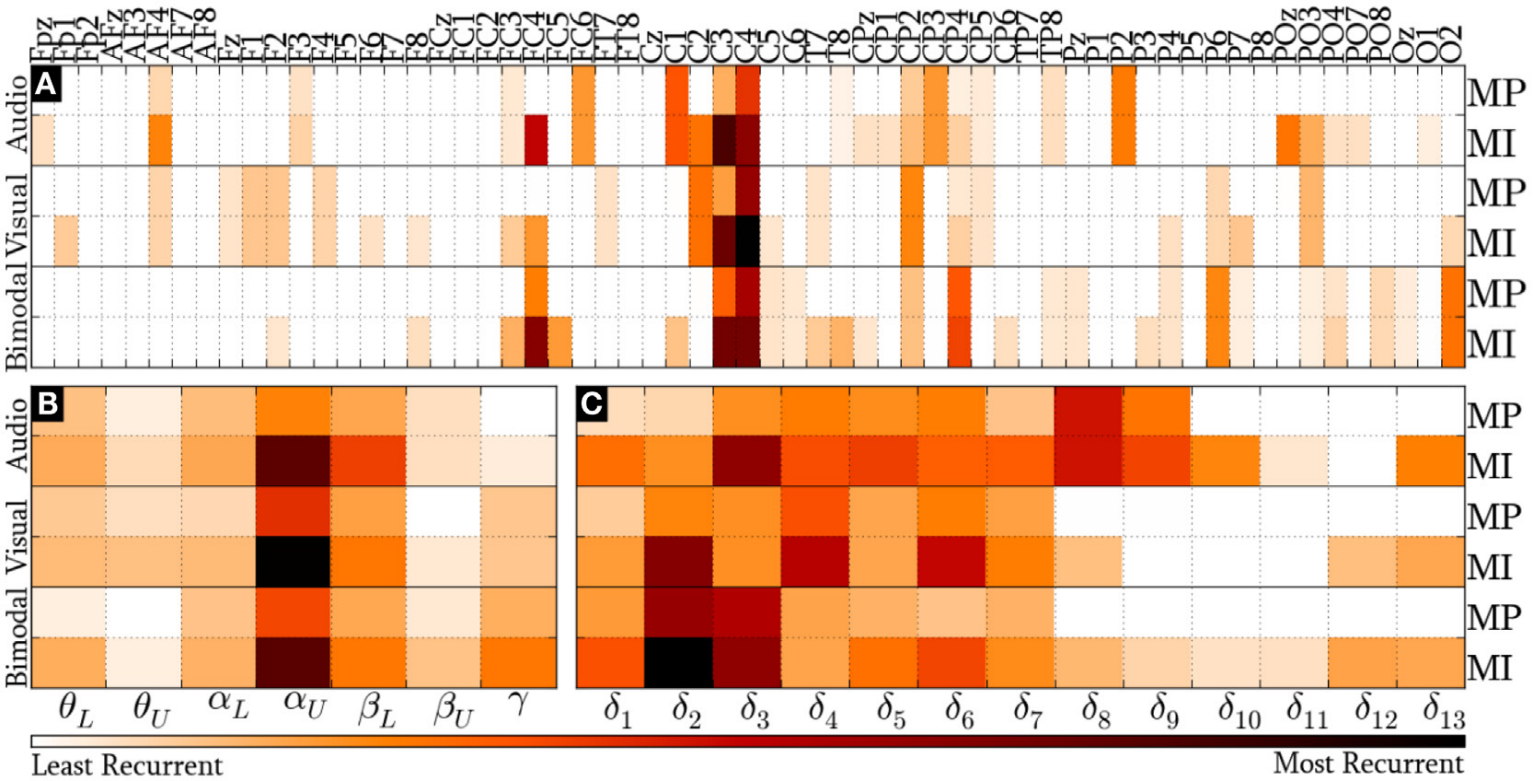
***Modal* distribution of the HQFVs that resulted from the all-embracing method**. The *modal* distribution is illustrated in three domains: **(A)** 61 recording sites, **(B)** 7 frequency bands, and **(C)** 9/13 time windows for MP/MI.

Figure 8 provides the *modal* distribution of the HQFVs over the following feature sources: (a) 61 recording sites, (b) 7 frequency bands, and (c) 9/13 time windows for MP/MI. With respect to the location domain, Figure 8A indicates that *modes* from the three SMs are distributed over about 40% of the feature sources in both control tasks. Specifically, *modes* from audio cues are distributed among 24 of the 61 recording sites. From those, 62% are on central areas, 25% are on parieto-occipital areas, and 13% are on frontal areas. *Modes* from visual cues are also distributed among 24 of the 61 recording sites. However, those are differently spread. Over half of them are distributed between frontal and parieto-occipital areas (33% and 21%, respectively), while less than half of them are related to central areas (46%). *Modes* from bimodal cues are distributed among 27 of the 61 recording sites. From those, 56% are on central areas, 37% are on parieto-occipital areas, and 7% are on frontal areas.

Figure 8B illustrates the prevalence of α_U_ band in the *modes* from the three SMs in both MP and MI. The figure also reveals the secondary but not insignificant role of β_L_ band. The *modal* distribution between θ_L_ and α_L_ bands is moderate for the three SMs, whereas that between θ_U_ and β_U_ bands is negligible for the three SMs. Furthermore, the *modal* distribution over γ band is considerable for bimodal cues. In all these cases, MI shows much higher tendencies than MP.

Last but not least, Figure 8C depicts the *modal* distribution in time, considering that MP only involved 9 of the 13 time windows. It can be seen from this figure that the *modes* from audio cues are spread across δ_1_ and δ_9_ (0–2500 ms), while those from visual and bimodal cues are spread across δ_1_ and δ_7_ (0–2000 ms). Particularly for MI control tasks, the *modes* from audio, visual, and bimodal cues strongly tend toward δ_3_ (500–1000 ms), δ_4_ (750–1250 ms), and δ_2_/δ_3_ (250–1000 ms), respectively. In addition, there is an unexpected *modal* tendency to δ_13_ for the three SMs in MI.

## Discussion

This paper set out with the aim of analyzing the cue effects on the discriminability of MP and MI control tasks. The analysis was carried out using two methods: standard and all-embracing. The standard method was based on feature sources, where the modulation of brain signals due to MP/MI is typically detected. For the all-embracing method, the scope of the standard method was extended by including a wider variety of feature sources, where not only motor activity is reflected. The analysis was limited to the HQFVs, i.e., the feature vectors that yielded the highest CAs during a DBI-FDA process. The following is a discussion of the most relevant results of the analysis.

### Classification Accuracy of the HQFVs

On the question of improving CA by using different cues, we found that there was no significant difference in the discrimination of MI-related control tasks triggered by three SMs: audio, visual, and bimodal. However, there was a significant increase in the CA of control tasks analyzed under the all-embracing method over those analyzed using the standard method. These findings demonstrated that an unbiased approach in location, frequency, and time leads to a better performance, but different cues do not make a difference. Although Scheel et al. (2015) suggested that different stimuli might improve the CA of the control tasks at hand, this study has been unable to demonstrate that. Nevertheless, it is a fact that more distinguishable EEG patterns are extracted from no MI-related sources. Possibly, other type of stimuli could improve the differentiation of MI-related control tasks. In accordance with the findings of Pfurtscheller and Neuper (2001) and Obermaier et al. (2003), control tasks are result of conscious and unconscious processes. As different stimuli may evoke different unconscious processes, more differentiable EEG patterns could be found. However, this needs further investigation.

Another important result was the analogous performances of MP and MI for the three SMs and the two methods. It is well established that both control tasks generate similar event-related oscillations, but the “no-go” signal accompanying MI is frequently overlooked (Krepki et al., 2007). An imaginary movement activates motor areas of the brain almost to the same extent as a real one, except for the visible contractions. This means that the neural commands for muscular contractions are blocked at some level of the motor system by an active inhibitory mechanism. This questions whether MI in motor-disabled people takes place like that in healthy ones, or it is rather a real movement process (Jeannerod, 2006). In addition, the use of MI as control task involves the development of an electromyographic detector so as to eliminate undesirable muscular activity un- or consciously triggered by healthy BCI users. Based on these two factors and given that both control tasks achieved analogous performances, MP may be a better option for BCI systems.

### Index of Dispersion of the HQFVs in Location, Frequency, and Time

For both methods, the distribution of the HQFVs over the available feature sources was much more even in location and time than in frequency. This indicates that the most gainful features for discriminating between left and right MIs mainly proceeded from the entire set of recording sites and the total duration of the control task, but only from a specific frequency band (α_U_). The finding is in agreement with that of other studies (Pfurtscheller and Neuper, 2001; Neuper et al., 2009) which showed that the correct discrimination between left and right started 250–500 ms after cue onset and where the most discriminating frequency band was the α_U_. With reference to the location domain, although this finding differs from some published studies (Ramoser et al., 2000; Leeb et al., 2006), it is consistent with those of Meckes et al. (2004) and Sepulveda et al. (2004). They suggested giving attention to non-motor locations, even when the mental task of interest was movement related.

The current study also found that the HQFVs tended to be more widely spread over the feature sources in the all-embracing method than in the standard method. It is worth mentioning that the inclusion of more feature sources increased the diversity of HQFVs. This result corroborate the ideas of Pfurtscheller and Neuper (2001) and Obermaier et al. (2003), who suggested that control tasks are result of the mental effort demanded by the control task (conscious process) and the sensory–cognitive processing of the cue (unconscious process).

### Modal Distribution of the HQFVs in Location, Frequency, and Time

The *modes* of the HQFVs fundamentally tended toward the expected sources (Pineda, 2005). These were the C3/C4 recoding sites and the α_U_/β_L_ frequency bands. The *modes* also revealed clear tendencies toward feature sources that reflected the nature of the cue in use. Before going on to discuss this further, it is necessary to mention that the *modal* tendencies were much greater in MI than in MP. The reason for this is not clear, but it may be due to the mental effort involved in each control task. MP is such an intention, whereas MI is a dynamic process that goes through many of the central phases of actual movements.

#### Standard Method

The *modes* from audio cues tended to the left hemisphere, where some language-related functions take place, whereas those from visual cues tended to the right hemisphere, where visual perception is processed (Kropotov, 2010). Being the bimodal cue, a composition of audio and visual cues, the corresponding *modal* tendency was to both hemispheres. This result suggests that the most discriminating features were defined not only by the MP/MI mechanisms but also by the sensory–cognitive processing of the cue in use. With respect to the frequency domain, the involvement of high frequency bands took importance successively in *modes* from audio, visual, and bimodal cues. This result may be related to previous work of Giannitrapani [whose work is cited in Kropotov (2010)], who found that high beta activity (21–33 Hz) increased when the stimulus structure complexity also increased. It is possible, therefore, that the cue complexity had played a significant role in the discrimination process of features as well. Regarding the time domain, the highest tendencies took more time (after the cue onset) to appear in *modes* from audio than from bimodal cues. Hence, it is also possible to hypothesize that more informative features were found earlier when a more direct cue was employed.

#### All-Embracing Method

The *modes* from audio cues mostly tended to central recording sites, where auditory evoked potentials are typically recorded (Proverbio and Zani, 2003), and to δ_3_ (500–1000 ms) time window, where brain rhythms normally respond to the recognition and/or retrieval of acoustic stimuli (Krause, 2006). On the other hand, the visual stimulation is registered around 200 ms post-stimulus as a response to modulations of alpha band rhythms over parieto-occipital areas and beta band rhythms over fronto-parieto-occipital areas (Kropotov, 2010; Andreassi, 2013). This may be a reason why *modes* from visual cues tended to fronto-parieto-occipital recording sites, α_U_ band, and δ_2_ (250–750 ms) time window. Finally, the *modes* from bimodal cues displayed a well-balanced distribution between central and fronto-parieto-occipital recording sites and between δ_2_ (250–750 ms) and δ_3_ (500–1000 ms). This finding confirms that bimodal stimuli require feature sources that are also required by audio and visual stimuli separately (Isoğlu-Alkaç et al., 2007). In the frequency domain, the remarkable tendency of these *modes* toward γ band accords with previous findings of Ward (2003), who found that the sensory decoding around 250 ms post-stimulus is reflected in modulation of γ band rhythms.

In the three SMs, one unanticipated finding was the minor role occupied by β_U_ band that is well-known as one of the major contributors in the discrimination process of MI activity. A possible explanation for the small contribution of this band is that neural desynchronization around 20 Hz has been considered as a harmonic response of desynchronization around 10 Hz, whereas the one around 16 Hz is an authentic response to motor activity (Pfurtscheller et al., 1996). Moreover, Pfurtscheller et al. (1999) found that the most discriminating frequency components throughout MI-related tasks were found within the α_U_ band in three of four subjects, while those were found within the β_U_ band only in one subject.

Lastly, it is worth noting the underlying tendency of *modes* from visual and bimodal cues toward the δ_9_ (2000–2500 ms) time window in the standard method. There was also another clear tendency of *modes* from the three SMs toward the δ_13_ (3000–3500 ms) time window in both methods. For visual and bimodal stimulation, we believe that gaze fixation at the screen center provoked by cues “left”/“right” could have driven the participants to anticipate the upcoming cue “start.” Similarly, the cue “start” appearance caused the cue “stop” expectation. For audio stimulation, once the participants had received the cue “start,” and owing to the likeness between increasing and decreasing tones (cues “start” and “stop,” respectively), the anticipation of the audio cue “stop” was likely to have arisen. This speculation is supported by the findings of Scherer et al. (2008), who found that the involuntary expectations for the approaching cues provoked false control commands during virtual navigation. Another interesting tendency of *modes* of the three SMs is toward δ_2_ (250–750 ms) and δ_3_ (500–1000 ms) time windows in the all-embracing method. These results are in agreement with the findings of Pfurtscheller et al. (2008), who showed that distinct short-lasting brain patterns appeared within a time window of about 500–750 ms after cue onset.

All these interpretations must be, however, taken with caution. More research on this topic need to be undertaken because these findings can only be conclusive in early training sessions. The effects observed in this study could diminish or vanish, either in further training sessions or in online applications. Another source of uncertainty is associated with the ambiguity of multivariate classifiers (such as FDA) to determine the brain regions, frequency, and time intervals where cognitive processes are reflected. Haufe et al. (2014) demonstrated that backward models (e.g., multivariate classifiers) combine information from different channels to separate the brain patterns of different classes. These models may, however, give significant weight to channels unrelated to brain processes of interest. By contrast, forward models (e.g., blind source separation) explain how the measure data are generated from the neural sources, providing a neurophysiological meaning of the outcomes. Furthermore, Haufe et al. showed that brain patterns were much smoother and covered more diverse cognitive-related areas, when those patterns were obtained via forward methods. These findings are of particular interest due the nature of our study. It seems that the present results are limited by the methods applied to select the features vectors. Possibly, by transforming the backward model in use (DBI-FDA process) into a forward model such as proposed Haufe et al. (selection of brain patterns according to the neurophysiological contribution of each EEG channel), a clearer feature distribution over unrelated MI sources could have been achieved.

### Implications on Neuroergonomics Research

This is a key issue for Neuroergonomics research because neural activity could not only be used to monitor the human mental state, but this might be also employed to control a system of interest. In fact, Myrden and Chau (2015) have suggested to develop a BCI system on the basis of an overt adaptation to keep user mental within the optimal region, and a covert adaptation that automatically adjusts BCI parameters according to such mental state.

An important application may be on driver modeling and vehicle simulation environments (Xu et al., 2015). These two areas of research have been of interest to develop driver assistance systems for safer driving and intelligent transportation. For example, EEG signals of a driver can be used to model the driver neuromuscular dynamics (Bi et al., 2015) and, thus, improving the performance of a driver simulator. Such EEG signals can also be employed to detect the fatigue (Wang et al., 2014) and level of attention (Wang et al., 2015) of the driver to activate the driver simulator and, hence, preventing driving accidents. Furthermore, the performance of the driver simulator can be improved by analyzing the human reaction to traffic cues such as car horn, direction indicators, and traffic lights. All of these cues produce specific EEG patterns on the driver brain signals as has been shown in this study.

## Conclusion

The findings of this study have provided a new understanding of how MI-related control tasks used to control a BCI system may become modified by their preceding cues. Although previous investigations have somehow studied the cue effects on MI-related control tasks; in this study, we have shown that the CA of those control tasks does not depend on the type of cue in use. Moreover, we found that the EEG patterns that best differentiate MI-related control tasks emerge from recording sites, frequency bands, and time windows well defined by the perception and cognition of the cue in use. An implication of this study is the possibility of obtaining different control commands that could be detected with the same accuracy. Since different cues trigger control tasks that yield similar CAs, and those control tasks produce EEG patterns differentiated by the cue nature, this leads to accelerate the brain–computer communication by having a wider variety of detectable control commands. This is an important issue for Neuroergonomics research because neural activity could not only be used to monitor the human mental state, but this might be also employed to control the system of interest.

## Conflict of Interest Statement

The authors declare that the research was conducted in the absence of any commercial or financial relationships that could be construed as a potential conflict of interest.

## Footnotes

^1^Available at https://github.com/LuzAlondra/BrainComputerInterfaces/tree/master/MI-based_BCIsystem

^2^Before undertaking the classification process, note that the features were normalized using normalization and standardization methods developed by *mlpy* (high-performance Python package for predictive modelling avaliable at http://mlpy.sourceforge.net/) developers, to avoid BP estimates in greater numeric ranges dominate those in smaller numeric ranges.
